# *PSG* and Other Candidate Genes as Potential Biomarkers of Therapy Resistance in B-ALL: Insights from Chromosomal Microarray Analysis and Machine Learning

**DOI:** 10.3390/ijms26157437

**Published:** 2025-08-01

**Authors:** Valeriya Surimova, Natalya Risinskaya, Ekaterina Kotova, Abdulpatakh Abdulpatakhov, Anastasia Vasileva, Yulia Chabaeva, Sofia Starchenko, Olga Aleshina, Nikolay Kapranov, Irina Galtseva, Alina Ponomareva, Ilya Kanivets, Sergey Korostelev, Sergey Kulikov, Andrey Sudarikov, Elena Parovichnikova

**Affiliations:** 1 National Medical Research Center for Hematology, 125167 Moscow, Russia; 2017.e.s.kotova@gmail.com (E.K.); patakh1997@mail.ru (A.A.); vasilieva.a@blood.ru (A.V.); uchabaeva@gmail.com (Y.C.); starsof1309@mail.ru (S.S.); dr.gavrilina@mail.ru (O.A.); immunophenotyping.lab@gmail.com (N.K.); irinagaltseva@gmail.com (I.G.); smkulikov@mail.ru (S.K.); dusha@blood.ru (A.S.); parovichnikova.e@blood.ru (E.P.); 2 Intelligent Systems in Management and Automation Department, Moscow Technical University of Communications and Informatics, 111024 Moscow, Russia; 3 “Genomed” Laboratory of Molecular Pathology, 115419 Moscow, Russia; a.ponomareva@genomed.ru (A.P.); dr.kanivets@genomed.ru (I.K.); korostelevsa@genomed.ru (S.K.)

**Keywords:** acute B-lymphoblastic leukemia (B-ALL), copy number alterations (CNAs), copy neutral loss of heterozygosity (cnLOH), genes, machine learning

## Abstract

Chromosomal microarray analysis (CMA) was performed for 40 patients with B-ALL undergoing treatment according to the ALL-2016 protocol to investigate the copy number alterations (CNAs) and copy neutral loss of heterozygosity (cnLOH) associated with minimal residual disease (MRD)-positive remission. Aberrations involving over 20,000 genes were identified, and a random forest approach was applied to isolate a subset of genes whose CNAs and cnLOH are significantly associated with poor therapeutic response. We have assembled the triple matched healthy population data and used that data as a reference, but not as a matched control. We identified a recurrent cluster of cnLOH in the 19q13.2–19q13.31 region, significantly enriched in MRD-positive patients (70% vs. 47% in the reference group vs. 16% in MRD-negative patients). This region includes the pregnancy-specific glycoprotein (*PSG*) gene family and the oncogene *ERF*, suggesting a potential role in leukemic persistence and treatment resistance. Additionally, we observed significant deletions involving 7p22.3 and 16q13, often as part of large-scale losses affecting almost the entire chromosomes 7 and 16, indicative of global chromosomal instability. These findings highlight specific genomic regions potentially involved in therapy resistance and may contribute to improved risk stratification in B-ALL. Our findings emphasize the value of high-resolution CMA in diagnostics and risk stratification and suggest that *PSG* genes and other candidate genes could serve as biomarkers for predicting treatment outcomes.

## 1. Introduction

Despite substantial advances in treatment strategies, including risk-adapted chemotherapy protocols, a considerable proportion of B-ALL patients still exhibit MRD-positive remission after initial therapy, which is a strong predictor of relapse and poor outcome [1,2]. The genomic underpinnings of this phenomenon remain incompletely understood, particularly in terms of structural chromosomal abnormalities, which may affect large gene regions and contribute to therapy resistance.

Chromosomal microarray analysis has emerged as a valuable method for detecting submicroscopic copy number variations (CNVs) across the genome with higher resolution than conventional karyotyping or FISH [3,4]. Identifying not only amplifications and deletions but also regions of cnLOH is essential, as all these types of aberrations can disrupt gene dosage, alter regulatory networks, or unmask recessive mutations, thereby influencing disease biology and treatment response [5,6]. While previous studies have identified recurrent CNVs associated with leukemogenesis [7], less attention has been paid to their relationship with treatment response, especially in the context of MRD. Moreover, it remains controversial whether such CNVs represent inherited predispositions or are acquired somatic events (CNAs), with some studies suggesting a continuum between germline and somatic alterations [8,9].

In this study, we applied CMA to a cohort of patients with B-ALL treated under the ALL-2016 protocol, aiming to identify CNAs associated with MRD-positive remission. Given the high dimensionality of the genomic data—encompassing over 20,000 genes—we employed machine learning techniques, specifically the random forest algorithm, to assess the relative importance of these CNAs in relation to treatment response. Random forest is well-suited for such analyses due to its robustness in handling large feature spaces and its ability to provide measures of feature importance, facilitating the identification of potential biomarkers [10,11]. By comparing the prevalence of significant CNAs in our patient cohort with a matched reference group of healthy individuals, we aimed to discern whether these aberrations are likely disease-related. Our findings reveal a set of candidate genes whose aberrations may play a role in resistance to therapy and offer new insight into the genetic architecture of poor response in B-ALL.

## 2. Results

CMA was performed for 40 randomly chosen newly diagnosed Ph-negative B-ALL patients who received treatment from 2019 to 2023 according to ALL-2016/ALL-2016m protocol and had available tumor DNA material at the onset of the disease. The median age was 30 (18–55). The male-to-female ratio was 22 (55%) to 18 (45%). According to the classification of the European Group for the Immunological Characterization of Acute Leukemias (EGIL), the following are true:

- The B-I variant was diagnosed in five patients, three of whom showed involvement of 11q23.
- The B-II variant was found in 35 patients, including 1 with t(1;19).

Refractory disease (defined as >5% blasts in the myelogram on Day 70 of therapy) was diagnosed in 3 (7.5%) of the 40 patients. One patient (2.5%) with a translocation involving the MLL gene locus at 11q23 showed a shift in immunophenotypic variant during phase I of induction therapy.

Bone marrow remission was achieved in 36 (90%) patients after completion of two phases of induction therapy under the ALL-2016/ALL-2016m protocols. MRD persistence was confirmed in 18 (50%) of the 36 patients. Relapse was diagnosed in three patients, all of whom were MRD-positive on Day 70 of therapy according to the ALL-2016 protocol. Allogeneic hematopoietic stem cell transplantation (allo-HSCT) was performed in eight patients: two with 11q23 involvement, two with primary refractory disease, one with t(1;19), one patient due to prolonged MRD persistence, and one patient after achieving a second remission.

Nine patients died: one case of early mortality; in two patients, a refractory relapse occurred after allo-HSCT; one patient died in remission due to infectious complications; and five patients developed a refractory relapse at various stages of treatment under the ALL-2016 protocol.

### 2.1. The Landscape of Gains, Losses, and cnLOH in B-ALL

**Figure 1:**
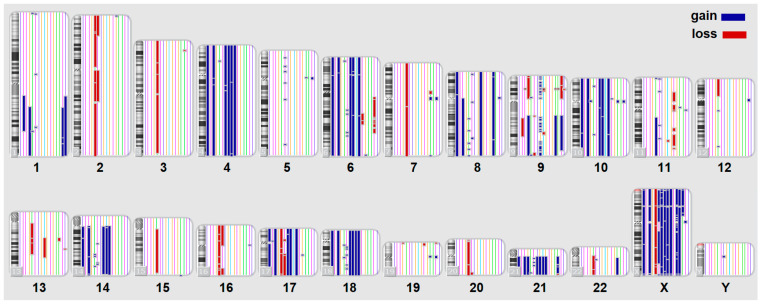
Loss and gain aberrations for B-ALL patients at the onset of the disease. Aberrations exceeding 500 Kbase are presented. Blue color for gain aberrations, red for loss aberrations.

**Figure 2:**
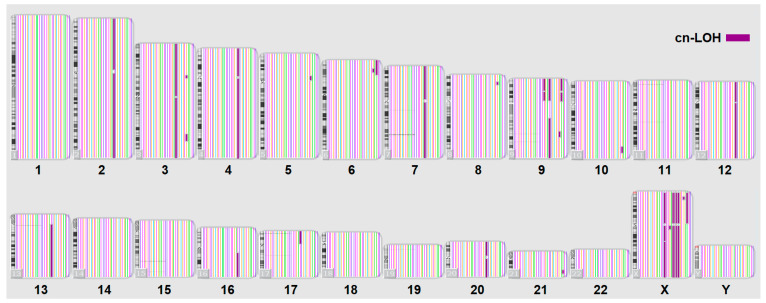
cnLOH for B-ALL patients at the onset of the disease. Aberrations exceeding 5000 Kbase are presented. Purple color for cnLOH.

In our study, CMA identified a total of 46,581 genomic aberrations across 40 adult B-ALL patients. These included both copy number gains and losses, as well as copy-neutral loss of heterozygosity (cnLOH), a category of structural variation that is often overlooked by conventional cytogenetic methods. The distribution of large-scale chromosomal gains and losses across all chromosomes is illustrated in Figure 1, while the landscape of cnLOH events is shown in Figure 2.

The vast number and diversity of these alterations reflect the high degree of interpatient genomic heterogeneity in adult B-ALL, which complicates risk stratification and the identification of actionable markers.

### 2.2. Aberrations Associated with MRD^+^ Remission

For the downstream analysis, we selected data from 35 adult B-ALL patients for whom complete clinical and cytogenomic information was available. Among them, 17 patients exhibited MRD-positive remission status following induction therapy. The dataset included 76,461 variables, each representing a specific gene and its type of aberration—whether it was a gain, loss, or cnLOH. We also have combined loss and loss mosaic types as loss. This gene-centric encoding enabled a comprehensive assessment of the relationship between specific genomic alterations and MRD status.

To identify genomic features most predictive of MRD^+^ status, we applied a random forest classifier—a robust ensemble learning method well-suited for high-dimensional biological data. One of the key outputs of this model is feature importance, which quantifies how much each variable contributes to improving the model’s prediction accuracy. In the context of decision trees, importance is typically derived from the reduction in impurity (e.g., Gini index) attributed to splits involving a particular feature across the entire forest.

**Figure 3:**
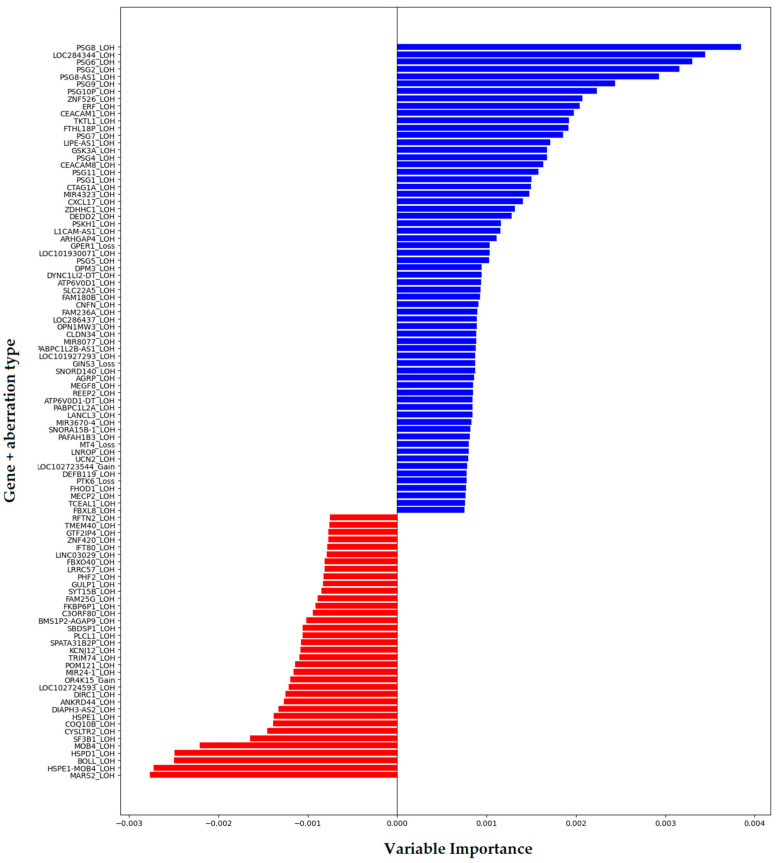
Top 100 genes with MRD remission-associated aberrations: blue color for MRD^+^ remission risk factors, red for favorable MRD aberrations.

Based on feature importance scores, we identified sets of genes whose aberrations were most strongly associated with MRD remission. The top 100 genes are shown in Figure 3, ranked by their relative importance to the classification task. The top features include a few known oncogenes and tumor suppressors, as well as lesser-known genes that may play a previously unrecognized role in therapy resistance. This highlights the potential of machine learning not only for predictive modeling but also for biomarker discovery in complex, heterogeneous diseases like B-ALL.

Importantly, we observed that some genomic aberrations were significantly enriched among MRD^+^ patients, suggesting a potential role in therapy resistance or disease persistence. In contrast, other aberrations were more frequently found in MRD-negative individuals, indicating a possible association with favorable early treatment response.

**Figure 4:**
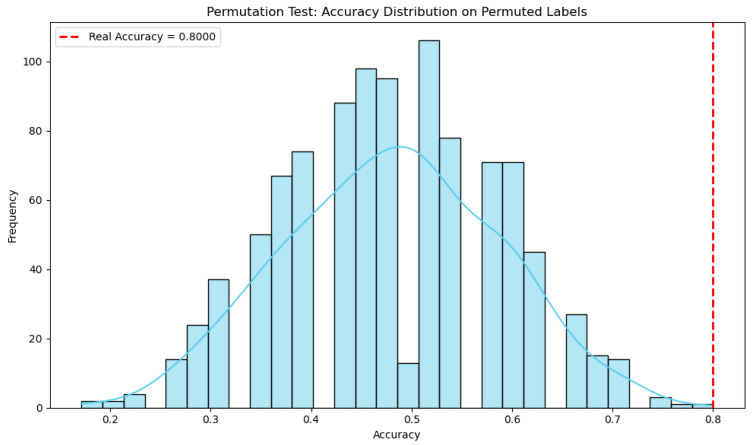
Permutation test: histogram of permuted model distribution and real accuracy red dot line.

To assess whether the classification performance of our model exceeds what could be expected by chance, we conducted a permutation test. Specifically, we trained a random forest classifier on the original data and computed its cross-validated accuracy. We then generated a null distribution by randomly permuting the class labels 1000 times, training a new model on each permuted dataset, and recording the resulting accuracies. Since these permutations break any real dependency between features and labels, the resulting distribution reflects the range of accuracies attainable under the null hypothesis of no association. The *p*-value was calculated as the proportion of permuted accuracies that exceeded the accuracy obtained on the original (unpermuted) data. This approach provides a non-parametric estimate of statistical significance and helps ensure that the observed performance is not simply due to overfitting or chance. In our case, the real accuracy was 0.80, while the entire null distribution lay below this value, resulting in a *p*-value of 0.001. This strongly suggests that the model’s performance is statistically significant and unlikely to be due to random chance. A visualization of the null distribution compared to the real accuracy is provided in Figure 4.

To further validate the findings obtained from the machine learning model, we conducted univariate statistical testing using Fisher’s exact test, followed by correction for multiple hypothesis testing. A full table containing the frequency of aberrations in the MRD^+^ and MRD^−^ groups and in the reference cohort, along with the *p*-values and odds ratios for the top genes, is provided in the Supplementary Table S1 (for *p* < 0.05). Several of the top-ranked genes identified by the random forest model also showed statistically significant differences in aberration frequencies between MRD^+^ and MRD^−^ groups, thereby reinforcing their potential biological and clinical relevance. We have discovered that among the top 200 most frequent unfavorable features, there are three clusters of genes with the same localization and aberration type: cnLOH in the 19q13.2-19q13.31 region, and mosaic loss in regions 7p22.3 and 16q13.

We identified a cluster of genes affected by cnLOH in the 19q13.2–19q13.3 region specifically in MRD^+^ patients. This region includes the *PSG* gene family, as well as the oncogene *ERF*, both of which may contribute to leukemic progression and treatment resistance. In addition, we observed significant aberrations involving genes located in 7p22.3 and 16q13. However, in most cases, these alterations were part of large-scale deletions involving nearly the entire chromosome 7 or 16, suggesting widespread chromosomal instability rather than focal gene loss.

An aggregated list of gene-level aberration frequencies in MRD^+^ versus MRD^−^ subgroups is provided in Table 1.

Standard cytogenetics did not detect these deletions because they were present only in a subset of cells and did not reach the detection threshold of conventional karyotyping. In most patients with deletions of chromosomes 7 or 16, mosaicism was observed—meaning the deletion was present in a subset of cells smaller than the overall blast population. This suggests tumor heterogeneity and the presence of a minor subclone harboring monosomy of chromosomes 7 and 16 in four MRD-positive patients. In one patient, a deletion of chromosome 16 was detected in all analyzed cells, while a deletion of chromosome 7 was present in 37% of cells, indicating the existence of a subclone with deletions of both chromosomes.

This concordance between multivariate model-based ranking and univariate frequency analysis underscores the robustness of our findings and suggests that these aberrations may serve as candidate biomarkers for risk stratification in adult B-ALL.

**Figure 5:**
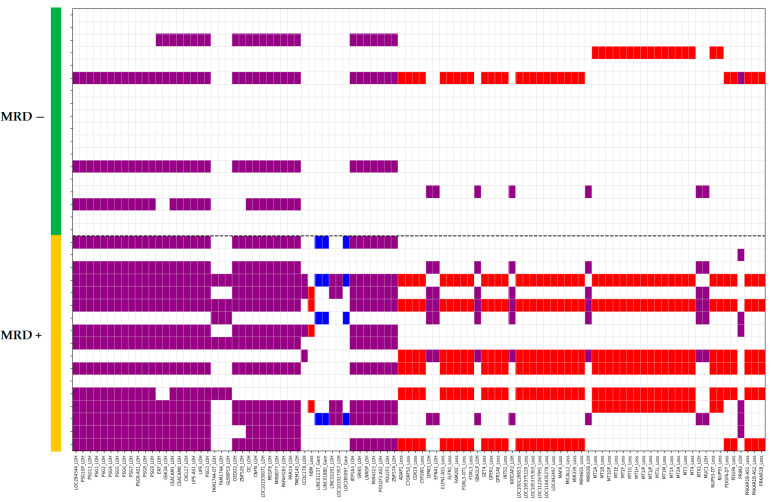
Heatmap of aberrations in the top 200 RF-ranked genes across 35 patients. Purple for cn-LOH, red for loss, blue for gain.

To further explore the distribution of the most informative aberrations across the entire cohort, we generated a heatmap visualizing the presence or absence of genomic alterations in the top-ranked genes (based on feature importance). The resulting heatmap, shown in Figure 5, highlights both shared and distinct genomic patterns among MRD^+^ and MRD^−^ remission.

The heatmap generated for the top-ranked genes across all patients revealed that aberrations in three gene clusters frequently co-occur. These findings support the hypothesis that *PSG* genes may serve as a central driver of treatment resistance in B-ALL, with other associated gene alterations potentially reflecting secondary or co-selected events.

**Figure 6:**
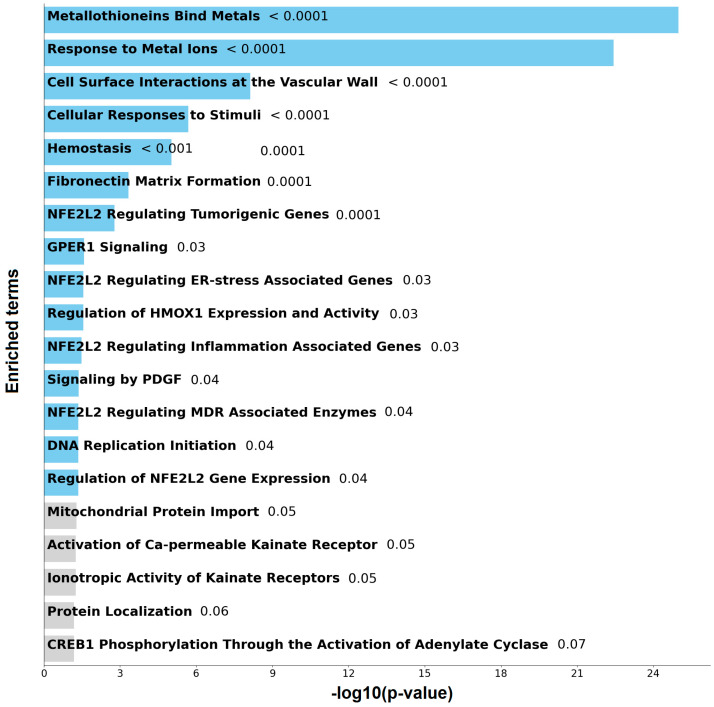
Bar chart of top enriched terms from the Reactome Pathways 2024 gene set library [12]. The top 20 enriched terms for the input gene set are displayed based on the −log10(*p*-value), with the actual *p*-value shown next to each term. The terms that have significant overlap with the input query gene set are colored in blue.

To further investigate the biological relevance of the top genes, we performed pathway enrichment analysis using the Reactome 2024 database [12] to identify signaling pathways in which these genes are involved. The top 20 enriched pathways, along with their associated *p*-values, are presented in Figure 6. Notably, the most significantly enriched pathways include Metallothioneins Bind Metals (*p* < 0.0001), Response to Metal Ions (*p* < 0.0001), and Cell Surface Interactions at the Vascular Wall (*p* < 0.0001), among others. A complete list of enriched pathways and the corresponding genes involved is provided in Supplementary Table S2.

The most enriched pathway, Metallothioneins Bind Metals, involves metallothionein factors: MT1A, MT1B, MT1E, MT1F, MT1G, MT1H, MT1M, MT1X, MT2A, MT3, MT4. These proteins play critical roles in metal ion homeostasis and detoxification, particularly in response to heavy metals like zinc and cadmium. Metallothionein factors are also central in the Response to Metal Ions pathway, reflecting their shared biological function in buffering oxidative stress and modulating metal-responsive signaling. The Cell Surface Interactions at the Vascular Wall pathway includes multiple pregnancy-specific glycoproteins (PSG1–PSG11) and adhesion molecules such as CEACAM1 and CEACAM8, suggesting a role in immune modulation and cell–cell interactions, particularly in the context of inflammation or tumor vascular microenvironments. Other enriched pathways include more general stress and signaling responses. For instance, Cellular Responses to Stimuli (*p* < 0.0001) encompasses factors such as PDGFA, GSK3A, PRKAR1B, and several metallothioneins, implicating them in diverse cellular adaptation mechanisms. The Hemostasis pathway (*p* < 0.0001), involving PSGs, CEACAMs, and PDGFA, suggests that some of the deregulated genes may also influence vascular integrity or coagulation-related processes.

Importantly, several pathways point toward regulation by *NFE2L2* (also known as *NRF2*), a master regulator of oxidative stress responses. Genes such as *MAFK*, a transcriptional partner of *NFE2L2*, appear in multiple related pathways, including *NFE2L2* Regulating Tumorigenic Genes, ER-Stress Associated Genes, Inflammation Associated Genes, and MDR Associated Enzymes. This highlights a potential role of the *NFE2L2/MAFK* axis in stress adaptation, drug resistance, and tumor progression.

### 2.3. Comparative Analysis with Population Reference Data

To evaluate whether the chromosomal aberrations associated with MRD-positive status in adult B-ALL patients reflect somatic, disease-specific events rather than common constitutional variants, we compared our findings with data from a cohort of 105 healthy individuals. These individuals underwent chromosomal microarray testing as part of preconception genetic screening or genetic counseling, and were not diagnosed with hematologic or oncologic diseases.

We constructed a triple-matched reference set by aligning individuals based on sex and age. The median age was 34 (18–58). This approach minimizes confounding and ensures that any differences in aberration frequencies can be attributed more confidently to disease status rather than population structure or technical variability. The use of matched references is known to substantially increase the statistical power and interpretability of genomic association studies, particularly when working with high-dimensional data. Methods for performing this matching and the associated statistical benefits have been well documented [13].

Tools developed specifically for focal CNA analysis, such as the FocalCall 1.4.0 package, highlight the importance of using matched references to enhance the detection of relevant copy number events and reduce false positives in high-dimensional genomic datasets [14].

**Figure 7:**
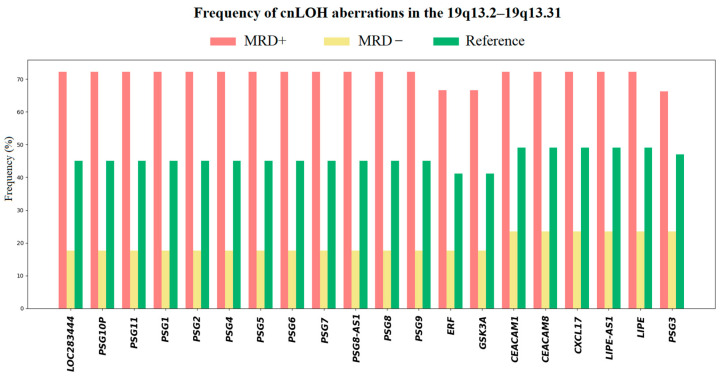
Frequency of 19q13.2–19q13.31 aberrations in patients and reference group: red color for MRD^+^ patients, yellow for MRD^−^ patients, green for reference group.

This approach allowed us to deduce which variants are likely disease-associated aberrations, strengthening the clinical relevance of our top candidate genes. We found that our top-ranked cnLOH aberrations in the 19q13.2–19q13.31 region are also present in the reference group, with an average frequency of 47% (Figure 7). Notably, these aberrations are significantly more prevalent among MRD-positive patients, occurring in approximately 70% of cases, highlighting a potential association with poor treatment response. Notably, aberrations in regions 7p22.3 and 16q13 were virtually absent in the relatively healthy cohort.

We also compared the size of aberrations, marker count, and gene count in MRD^+^ group and reference group. There is no difference between these parameters (Table 2).

These findings emphasize the importance of comparing patient data with reference cohorts when prioritizing candidate genomic markers, particularly in diseases with highly heterogeneous karyotypic profiles such as adult B-ALL. The virtual absence of most candidate gene aberrations in the healthy cohort reinforces their potential relevance to leukemogenesis and MRD persistence.

## 3. Discussion

In our previous study, we focused on known genetic drivers of B-ALL and their associations with MRD positivity, analyzing chromosomal microarray data with an emphasis on selected candidate genes of established relevance [15]. While this targeted approach yielded important insights, it left much of the genomic landscape unexplored. In the present study, by leveraging the full spectrum of CMA data—including 76,461 gene-level features and various types of copy number alterations—we adopted a machine learning framework to uncover novel patterns and associations. Our goal was to identify copy number aberrations associated with MRD positivity after induction therapy, which is a well-established prognostic factor for relapse and adverse outcomes in B-ALL [16,17]. Through a high-dimensional, machine-learning-based analysis, we identified a set of candidate genes whose involvement via copy number changes may contribute to therapy resistance and MRD persistence.

CMA allowed us to comprehensively detect a wide range of structural variants, including not only gains and losses but also cnLOH, which can be missed by conventional cytogenetics [18,19]. The ability of CMA to detect a wide range of aberration types with high resolution underscores its utility in the comprehensive genomic profiling of hematologic malignancies [20]. This approach revealed 76,461 total aberrations across 40 patients, emphasizing the complexity and heterogeneity of leukemia-associated genomic alterations (Figure 1 and Figure 2). Identifying clinically meaningful patterns within this high-dimensional landscape necessitated the use of machine learning. In our study, random forest modeling enabled the selection of features (genes and aberration types) most strongly associated with MRD-positive status, even in the presence of >20,000 variables. Given this complexity, it is critical to uncover meaningful patterns within this high-dimensional genomic data. Data-driven approaches, such as machine learning, can be instrumental in identifying subsets of aberrations associated with clinical outcomes, such as minimal residual disease (MRD) status, thereby contributing to the development of more precise prognostic tools and personalized treatment strategies [21].

Our results indicate that the classifier achieved a cross-validated accuracy of 0.80 on the original, unpermuted data, while the distribution of accuracies under label permutation remained entirely below this value (*p* = 0.001). This suggests that the model was able to capture a statistically significant relationship between features and labels, rather than fitting to noise or artifacts. Given the relatively small sample size, such validation is especially important, as high-capacity models like random forests are prone to overfitting. Permutation testing provides a rigorous, non-parametric method to assess the likelihood of obtaining a given performance metric under the null hypothesis of no association [22]. Unlike traditional statistical tests that assume specific data distributions, permutation testing adapts directly to the structure of the data, making it well-suited for complex, high-dimensional, or small-sample settings commonly encountered in biomedical and behavioral studies [23].

**Figure 8:**
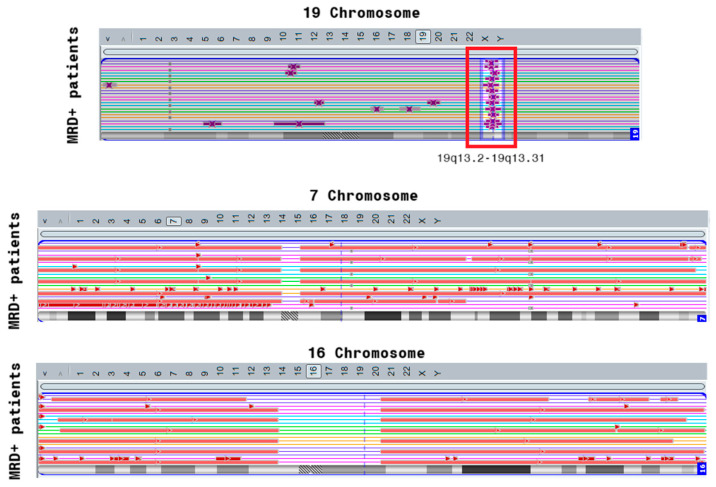
Localization of top genes associated with MRD-status of B-ALL. Purple for cn-LOH, red for loss aberrations.

The final top gene list included both known cancer-associated genes (e.g., *ERF*) and genes with previously unknown relevance to leukemia, such as *PSG* genes. The relevance of most aberrations involving these genes as risk factors for MRD^+^ remission was supported not only by the random forest’s feature importance scores (Figure 3), but also by Fisher’s exact test with multiple comparison correction, confirming statistical enrichment of aberrations in MRD-positive patients. The top-ranked genes identified in association with MRD positivity were mostly distributed across three chromosomes (Figure 8).

PSGs are a family of immunoglobulin superfamily proteins predominantly expressed by placental trophoblasts. While their physiological role is mainly in immunotolerance during pregnancy, recent research has highlighted their involvement in cancer progression, immune modulation, and potentially in chemotherapy resistance.

In several solid tumors, *PSG*s—especially *PSG1* and *PSG9*—are upregulated and contribute to a protumorigenic microenvironment. In colorectal cancer (CRC), *PSG9* has been shown to promote angiogenesis through interaction with *SMAD4*, leading to enhanced nuclear retention of *SMAD4* and activation of angiogenesis-related genes such as *VEGFA* and *PDGF-AA* [24].

In cervical cancer, *PSG1* gene amplification and overexpression have been observed. Its increased levels were associated with an immunosuppressive tumor microenvironment, as PSG1 upregulates cytokines such as IL-10 and TGF-β, which can facilitate immune escape [25].

In pancreatic ductal adenocarcinoma (PDAC), PSG1 expression has been detected in tumor tissues. Its subcellular localization (cytoplasmic vs. nuclear) was found to correlate with patient outcomes, suggesting a potential prognostic role [26].

Although direct links between PSGs and chemoresistance are still under investigation, their known roles in angiogenesis and immune modulation may indirectly support tumor survival in the presence of cytotoxic agents. By promoting regulatory T-cell differentiation via TGF-β signaling, PSGs may create an immunosuppressive niche that allows cancer cells to evade both immune surveillance and therapeutic attacks [27].

The role of PSGs in hematologic malignancies is less defined, but given their immunomodulatory function, it is plausible they contribute to disease persistence or relapse in oncohematology. For instance, the induction of FoxP3+ regulatory T-cells through TGF-β1 by PSGs could impair antitumor immunity, a mechanism relevant in leukemias and lymphomas where immune evasion plays a key role [28].

PSGs are attractive immunotherapeutic targets because of their limited expression in normal adult tissues and overexpression in tumors. Therapeutic strategies aimed at blocking PSGs or their downstream effects could inhibit tumor angiogenesis and restore immune responsiveness.

The newest 2025 study titled by Jung Hun Oh [29] and colleagues investigates the role of PSGs in lung adenocarcinoma (LUAD). The study found that elevated expression of PSG genes—particularly *PSG3*, *PSG7*, and *PSG8*—is significantly associated with poorer overall survival. These findings were consistent across analyses of The Cancer Genome Atlas (TCGA) and were validated using data from the Clinical Proteomic Tumor Analysis Consortium (CPTAC). Pathway analysis indicated that high PSG expression in female patients correlates with alterations in the KRAS signaling pathway. Given that KRAS mutations are common in LUAD and associated with aggressive tumor behavior, this link suggests that PSGs may contribute to tumor progression through modulation of KRAS-related pathways.

PSGs are known to regulate immune responses during pregnancy, promoting tolerance to the fetus. In the context of cancer, their expression may similarly suppress anti-tumor immunity, facilitating tumor immune evasion. This immunosuppressive effect could be particularly detrimental in female patients, potentially due to interactions with sex hormones or differences in immune system dynamics.

Deletions in the short arm of chromosome 7, particularly 7p22.3, have been identified in various hematologic malignancies, including B-ALL. Although more commonly associated with myeloid malignancies, recent genomic profiling studies in B-ALL have revealed recurrent submicroscopic deletions at 7p22.3, encompassing genes involved in DNA repair, apoptosis, and lymphoid development [30].

The *IKZF1*-like gene family is frequently disrupted in this region, leading to altered transcriptional regulation of lymphoid differentiation and contributing to resistance to corticosteroids and other chemotherapeutic agents [31]. In pediatric B-ALL, 7p22.3 deletion has been associated with minimal residual disease (MRD) positivity post-induction, which is a strong predictor of relapse [32].

The 7p22.3 region includes several candidate tumor suppressor genes such as *USP42*, a ubiquitin-specific protease involved in p53 stabilization. Loss of *USP42* may lead to impaired p53-mediated apoptosis and reduced chemosensitivity [33].

Our approach to analyzing the association between the “gene-event” marker and therapy response highlights the deletion of genes in the 16q13 region, which encode metallothioneins (MT1A, MT1B, MT1DP, MT1E, MT1F, MT1G, MT1H, MT1IP, MT1JP, MT1L, MT1M, MT1X, MT2A, MT3, MT4, NUP93-DT, NUP93), as one of the most significant markers. Metallothioneins (MTs) are small, cysteine-rich proteins that play a key role in metal homeostasis, protection against heavy metal toxicity, DNA damage, and oxidative stress. In humans, there are four main isoforms (MT1, MT2, MT3, MT4), encoded by genes on chromosome 16q13, with MT1 comprising eight functional subtypes. MTs are involved in carcinogenesis by influencing tumor growth, progression, and therapy resistance. Their expression varies depending on tumor type, differentiation stage, mutations, and environmental factors. Differences in the expression of specific MT isoforms may be used for cancer diagnosis and treatment.

The review by Si et al. [34] discusses the mechanisms through which MTs influence tumor growth, angiogenesis, metastasis, microenvironment remodeling, immune evasion, and drug resistance. It emphasizes the potential of MTs as biomarkers for diagnosis and prognosis and explores approaches for targeted cancer therapy through modulation of MT isoforms. In particular, it notes decreased MT expression in acute leukemia, which is significantly associated with clinical outcome.

Another study focuses [35] on the dysregulation of MT1 metallothionein subtypes in TCF3::PBX1 pre-B-cell acute lymphoblastic leukemia (ALL). The goal of the study was to identify genes whose regulation is specifically disrupted by the TCF3::PBX1 translocation. It was shown that the expression of metallothioneins is significantly reduced.

Additionally, MT3, a tumor suppressor gene, is frequently inactivated in pediatric AML through promoter hypermethylation [36].

However, we found very limited information regarding the association of 16q13 deletion with clinical outcomes in B-ALL. We found only one mention of a 16q13 deletion between positions 57,275,940 and 57,331,381 (two OMIM genes are located in this region: ADP-ribosylation factor-like 2 binding protein (ARL2BP) and plasmolipin (PLLP)) in young adults with B-ALL [37].

In our study, the 16q13 deletion is not a standalone event in most patients. Instead, the loss of the 16q13 locus occurs as a result of the loss of the entire chromosome 16 (see Figure 8).

Deletions of 7p22.3 and 16q13 are newly identified markers of poor prognosis and therapy resistance in B-ALL. Their presence may indicate genomic instability, defective apoptosis, and impaired immune signaling, which are critical for effective leukemia clearance. Recognizing these cytogenetic abnormalities may aid in developing risk-adapted treatment strategies and new therapeutic approaches.

Altogether, our findings underscore the potential of CMA data, when combined with machine learning and matched-reference comparisons, to uncover novel genomic predictors of treatment response in adult B-ALL. Further functional validation of the implicated genes is warranted, especially in the context of PSG-driven regulatory networks. Our study also highlights the value of integrating multiple types of genomic variation (e.g., cnLOH, deletions, duplications) and supports a broader implementation of high-resolution CMA in adult leukemia diagnostics and risk stratification.

## 4. Materials and Methods

The study cohort consisted of 40 adult patients diagnosed with B-cell acute lymphoblastic leukemia (B-ALL), all of whom were treated under the ALL-2016 protocol. Inclusion criteria required that patients had measurable disease and underwent induction chemotherapy, with post-induction bone marrow samples collected for analysis. Since there is very limited information on population-level frequencies of LOH in public repositories, we turned to the local repository of the “Genomed” laboratory. Additionally, 105 healthy individuals were included as a reference cohort. These individuals underwent chromosomal microarray analysis (CMA) as part of preconception genetic counseling and were matched for sex and age. Written informed consent was obtained from all participants, and the study was approved by the local ethics committee.

Genomic DNA was extracted from bone marrow samples of B-ALL patients using standard extraction protocols [38]. All patients included in the protocol underwent immunophenotyping, cytogenetic and molecular tests of bone marrow samples at the onset of the disease. MRD was assessed at the end of induction (day 70) using 6- or 10-color flow cytometry of the bone marrow specimens.

CMA was conducted using the CytoScan™ HT-CMA 96F array platform (Thermo Fisher Scientific, Santa Clara, CA, USA) following the manufacturer’s recommended protocol. Experimental procedures were carried out at the Molecular Pathology Laboratory “Genomed” (Moscow, Russia). Genomic DNA was extracted from bone marrow samples collected from B-ALL patients prior to initiation of therapy. Each sample met quality control thresholds, with DNA input ranging from 100 to 200 ng and an A260/A280 ratio of ≥1.8. A reference male DNA sample of matched concentration (Thermo Fisher Scientific, USA) was used as control. Data acquisition and analysis were performed using the Multi Sample Viewer Software (v.1.1.0.11) and Chromosome Analysis Suite (ChAS v.4.3.0.71) provided by the manufacturer. At the pre-analytical stage of the study, DNA quality was assessed. The following quality criteria were established for inclusion in the analysis: non-degraded DNA, fragment length of at least 10,000 base pairs on electrophoresis, concentration of at least 3 ng/μL, and a minimum volume of 30 μL. For LOH detection, the marker-count threshold was set at 50. The validity of LOH regions was evaluated using the ChAS software. The marker-count threshold was also set at 50 for duplications and 20 for deletions. The validity of CNVs was determined based on the Mean log2Ratio value: greater than 0.25 or less than −0.25.

**Figure 9:**
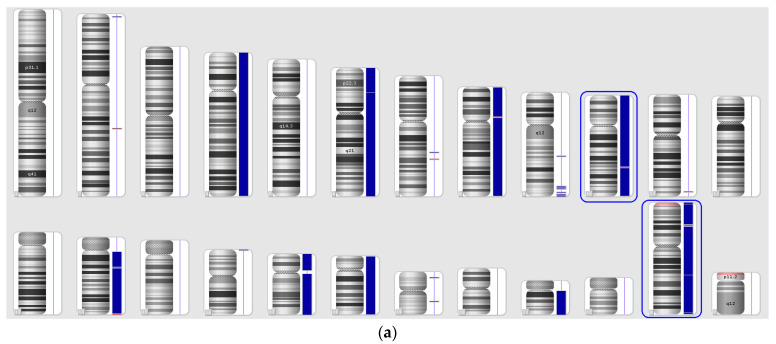

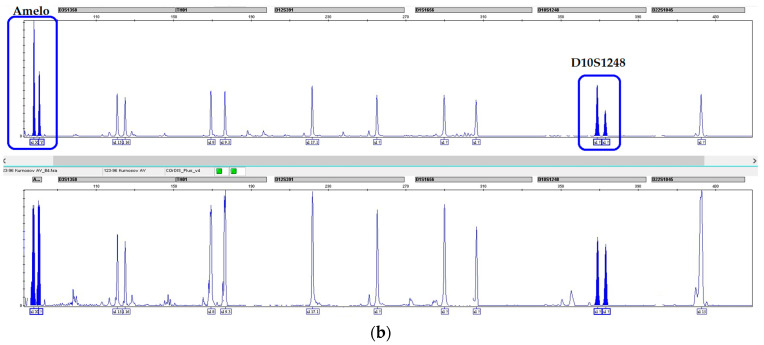
An example of verification of CMA-identified molecular karyotype aberrations (**a**) using STR analysis of paired tumor and normal DNA samples from a patient. (**b**) Duplications of chromosomes 10 and X are evidenced by allelic imbalance of the STR markers D10S1248 and Amelogenin (highlighted with blue frames).

All DNA samples were verified using the STR method [39], and the detected CMA-selected aberrations were confirmed by allelic imbalance relative to the STR profile of matched non-tumor tissue samples from each patient (Figure 9).

STR profiles for each sample were assessed by PCR with primers to 19 STR loci and amelogenin locus available in COrDIS Plus multiplex kit (Gordiz Ltd., Moscow, Russia). Following markers were studied: D1S1656 (locus 1q42), D2S441 (2p14), D3S1358 (3p21.31), D5S818 (5q23.2), D7S820 (7q21.11), D8S1179 (8q24.13), D10S1248 (10q26.3), D12S391 (12p13.2), D13S317 (13q31.1), D16S539 (16q24.1), D18S51 (18q21.33), D21S11 (21q21.1), D22S1045 (22q12.3), CSF1PO (5q33.1), FGA (4q31.3), SE33 (6q14), TH01 (11p15.5), TPOX (2p25.3), VWA (12p13.31), amelogenin X (Xp22.1–22.3), and amelogenin Y (Yp11.2). For fragment analysis of PCR products, a Nanophore-05 genetic analyzer (Institute of Analytical Instrumentation, Saint Petersburg, Russia) was used. STR profiles were then analyzed using GeneMapper Software (v. 4-0).

To identify key genomic features associated with MRD positivity, we employed a random forest model using Python 3.12. The scikit-learn library (version 0.24.0) was used to implement the model. Feature selection was performed using the RandomForestClassifier with 10-fold cross-validation (cross_val_score function). The top 10 genes most strongly associated with MRD positivity were selected based on feature importance scores. Statistical analysis of the feature importance was performed using Fisher’s exact test with multiple comparison corrections, using the statsmodels package (version 0.12.2). Heatmap analysis and clustering of aberrations were performed using seaborn (version 0.11.2) and matplotlib (version 3.4.3) libraries in Python.

To validate the disease-specific nature of the identified genomic alterations, we compared the aberrations found in the B-ALL patients to those observed in the healthy reference cohort. We specifically examined the top 200 genes identified from the random forest analysis for aberrations in the reference group, and statistical significance was assessed using Fisher’s exact test.

There are several limitations to our study that should be acknowledged. In the first part, the primary endpoint was treatment response, focusing on the distinction between responders and non-responders among B-ALL patients. In the second part, we expanded our analysis by incorporating data from a healthy population to compare the genomic profiles of ALL patients with those of individuals without hematological malignancies. However, the definition of the “healthy reference group” is not entirely aligned with the clinical endpoints of the patient cohort. While the patient analysis was based on therapeutic response, the comparison with healthy subjects reflects a broader contrast between disease and non-disease states. As such, the reference group should be interpreted as a reference population rather than a strictly matched reference in terms of clinical outcomes.

## 5. Conclusions

Our findings highlight a distinct genomic signature associated with MRD-positive B-ALL. The recurrent cnLOH in the 19q13.2–19q13.31 region—encompassing the *PSG* gene family and *ERF* oncogene—may contribute to leukemic cell survival and resistance to therapy. Furthermore, the frequent detection of broad deletions involving chromosomes 7 and 16, including critical regions 7p22.3 and 16q13, suggests a pattern of chromosomal instability linked to adverse clinical outcomes. These aberrations may serve as important biomarkers for refining prognostic models and guiding treatment strategies in B-ALL. Further functional validation is needed to explore the role of these genes in therapy resistance.

## Figures and Tables

**Table 1:** Distribution of aberrant events most strongly associated with MRD status in B-ALL cohort.

Localization	Genes	Event Type	MRD^+^ (*n* = 17)	MRD^−^ (*n* = 18)
*19q13.2-19q13.31*	*PSG10P, PSG11, PSG1, PSG2, PSG4, PSG5, PSG6, PSG7, PSG8-AS1, PSG8, PSG9*, etc.	LOH	13	3
*7p22.3*	*MAFK, MICALL2, MIR339*, *MIR4655, PDGFA-DT*, *PDGFA, PRKAR1B-AS1, ADAP1, C7ORF50*, *COX19, CYP2W1*, etc.	Loss	6	1
*16q13*	*MT1A, MT1B, MT1DP, MT1E, MT1F, MT1G, MT1H, MT1IP, MT1JP, MT1L, MT1M, MT1X, MT2A, MT3, MT4, NUP93-DT, NUP93*	Loss	6	1

**Table 2:** Characteristics of 19q13.2–19q13.31 aberrations in MRD^+^ B-ALL cohort and reference group.

Parameter	Size (kb) (MRD^+^/ref)	Number of SNP Markers	Gene Count
min	1045.83/1013.01	89/94	22/23
max	1949.01/2192.13	313/456	64/69
median	1428.68/1479.25	195/224	41/45

## Data Availability

Data are contained within the article and Supplementary Materials.

## Supplementary Materials

The following supporting information can be downloaded at: https://www.mdpi.com/article/10.3390/ijms26157437/s1.

## Abbreviations

The following abbreviations are used in this manuscript:

CMA	Chromosomal microarray
B-ALL	B-cell acute lymphoblastic leukemia
CNAs	Copy number alterations
cnLOH	Copy neutral loss of heterozygosity
MRD	Minimal residual disease
CNVs	Copy number variations
allo-HSCT	Allogeneic hematopoietic stem cell transplantation
PSG	Pregnancy-specific glycoprotein
